# The C-Terminal Domain of CENP-C Displays Multiple and Critical Functions for Mammalian Centromere Formation

**DOI:** 10.1371/journal.pone.0005832

**Published:** 2009-06-08

**Authors:** Stefania Trazzi, Giovanni Perini, Roberto Bernardoni, Monica Zoli, Joseph C. Reese, Andrea Musacchio, Giuliano Della Valle

**Affiliations:** 1 Department of Biology, University of Bologna, Bologna, Italy; 2 Department of Biochemistry and Molecular Biology, Center for Eukaryotic Gene Regulation, Pennsylvania State University, University Park, Pennsylvania, United States of America; 3 Department of Experimental Oncology, European Institute of Oncology, Milan, Italy; University of Munich and Center of Integrated Protein Science, Germany

## Abstract

CENP-C is a fundamental component of functional centromeres. The elucidation of its structure-function relationship with centromeric DNA and other kinetochore proteins is critical to the understanding of centromere assembly. CENP-C carries two regions, the central and the C-terminal domains, both of which are important for the ability of CENP-C to associate with the centromeric DNA. However, while the central region is largely divergent in CENP-C homologues, the C-terminal moiety contains two regions that are highly conserved from yeast to humans, named Mif2p homology domains (blocks II and III). The activity of these two domains in human CENP-C is not well defined. In this study we performed a functional dissection of C-terminal CENP-C region analyzing the role of single Mif2p homology domains through in vivo and in vitro assays. By immunofluorescence and Chromatin immunoprecipitation assay (ChIP) we were able to elucidate the ability of the Mif2p homology domain II to target centromere and contact alpha satellite DNA. We also investigate the interactions with other conserved inner kinetochore proteins by means of coimmunoprecipitation and bimolecular fluorescence complementation on cell nuclei. We found that the C-terminal region of CENP-C (Mif2p homology domain III) displays multiple activities ranging from the ability to form higher order structures like homo-dimers and homo-oligomers, to mediate interaction with CENP-A and histone H3. Overall, our findings support a model in which the Mif2p homology domains of CENP-C, by virtue of their ability to establish multiple contacts with DNA and centromere proteins, play a critical role in the structuring of kinethocore chromatin.

## Introduction

Proper chromosome segregation during cell divisions depends on a specialized chromosomal site, the centromere. This nucleo-proteinaceous element performs key functions in all eukaryotes from yeast to human. The structural organization of the centromere is generally multilayered and consists of a pairing domain that maintains the cohesion between sister chromatids, a central domain that contains specific centromeric DNA, and the kinetochore, the DNA/protein complex which provides the attachment site for spindle microtubules and regulates the movement of chromosomes towards the spindle poles [1]. Failure in any of these processes results in chromosome loss and gain leading to the formation of aneuploid cells. In most organisms, centromeres are constituted by large arrays of repeats known as satellite sequences (alpha satellite in humans). These DNA sequences direct the assembly of kinetochore proteins and are strikingly divergent between even closely related species. On the other hand, numerous kinetochore proteins have been identified in both human and model organisms and found to be very conserved during evolution [2]–[5]. Therefore, understanding how these highly conserved proteins assemble onto divergent satellite DNA to form functional centromeres remains one of the key problems in chromosome biology. Mammalian centromeres contain mega bases of repetitive satellite DNA. This is organized into specialized chromatin consisting of nucleosomes in which histone H3 is replaced by CENP-A, an H3-like variant. This protein is composed of a variable N-terminal region and a conserved C-terminal region carrying a histone-fold domain similar to that of histone H3 [6]. Since CENP-A depletion reduces fidelity of chromosome segregation and causes mislocalization of various kinetochore proteins [7], [8], it is believed that this protein may hierarchically recruit other centromere and kinetochore components to generate a high-order chromatin structure required for the formation of the inner kinetochore surface (for recent reviews see: [9], [10]).

**Figure 1:**
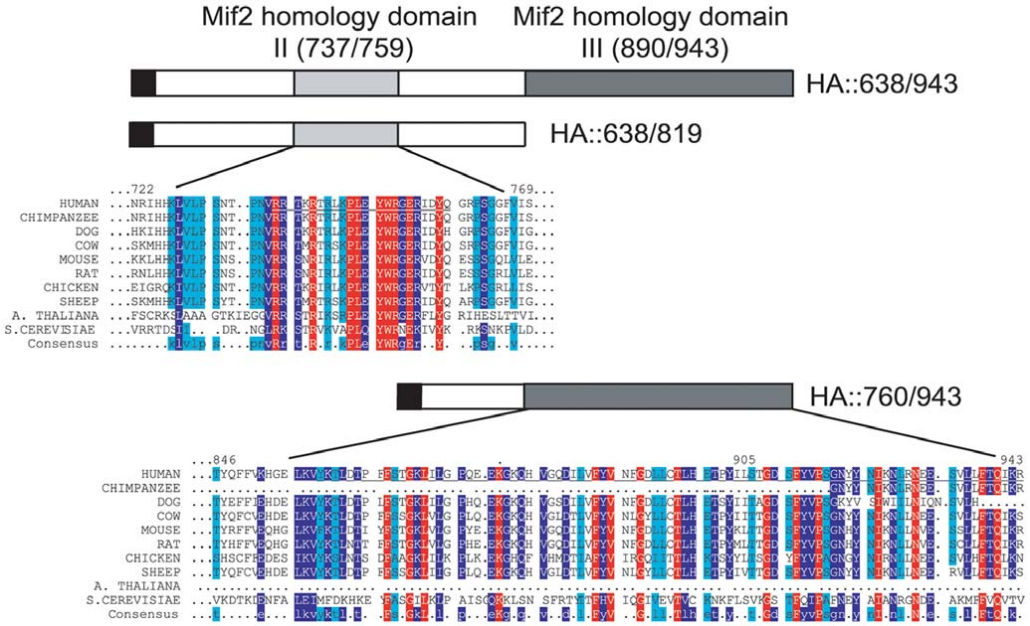
Sequence similarity among Mif2p homology domains of CENP-C orthologs. Amino acid sequences of human CENP-C Mif2p homology domains II and III, contained in the HA::638/819 and HA::760/943 constructs respectively, are compared to those of orthologous CENP-C proteins. Mif2p homology domains II and III are indicated in light grey and dark grey, respectively. Percent conservation is represented as follows: 100% conservation (red), 90% conservation (blue), 80% conservation (cyan); 70% conservation or less (white). Multiple sequence alignments of the CENP-C protein families were built with ClustalW, Multialin version 5.4.1 and T-Coffee and edited by hand [64]–[66]. Accession Numbers: Human CENP-C (GenBank M95724); Chimpanzee CENP-C (GenBank XM517266-7); Dog CENP-C (GenBank XM532388); Cow CENP-C (GenBank XM598358); Mouse CENP-C (GenBank U03113); Rat CENP-C (GenBank AAU04621.1); Chicken CENP-C (GenBank BAA24110.1); Sheep CENP-C (GenBank AAA79099.1); *A. thaliana* CENP-C (GenBank AAU04629.1); *S. cerevisiae* Mif2p (GenBank NP012834.1).

CENP-C is another essential kinetochore protein that localizes to the inner kinetochore plate [11] and associates with the alpha satellite DNA [12], [13]. Like CENP-A, CENP-C is involved in the assembly of kinetochores and in the correct segregation of sister chromatids [11], [14]–[17]. Moreover, this protein is a marker of functional centromeres and is present in conventional centromeres, neocentromeres and only in the active centromere of dicentric chromosomes [1], [18]–[22]. CENP-C contains two distinct domains, one in the central region and another in the C-terminal region; both can target the centromere and bind alpha satellite DNA *in vivo* [13]. Notably, comparative analysis of the CENP-C homologues isolated from other species shows that the central and C-terminal region of the human CENP-C display different degrees of conservation [23]. Particularly, while the central domain is poorly conserved, the C-terminal domain contains two regions that are highly conserved from yeast to mammals, suggesting that such regions might be preserved during evolution to exert critical centromere functions (Figure 1) [23]. Based on their degree of conservation with the yeast Mif2 protein, the two regions have been named Mif2p homology domain II and III [24]. The Mif2p homology domain II (aa 737/759 of human CENP-C), also called CENP-C motif [23], is present in all CENP-C homologues, though its specific function has not been defined. The Mif2p homology domain III (aa 890/943 of human CENP-C) appears to be required, at least in vitro, for CENP-C dimerization [25] and shares significant similarity with a protein motif that appears to be responsible for the oligo-multimerization of Cupin proteins [26]. Despite these findings, the specific contribution of the Mif2p homology domains of CENP-C to the assembly and function of centromeres is still unclear. In this study, by means of a multidisciplinary approach we show that these domains display multiple functions ranging from the ability to associate with centromeric DNA, to form higher order structures like homo-dimers and homo-oligomers, and to mediate interaction with CENP-A and histone H3.

## Results

### The Mif2p homology domain II of CENP-C targets centromeres and binds the alpha satellite DNA in vivo

**Figure 2:**
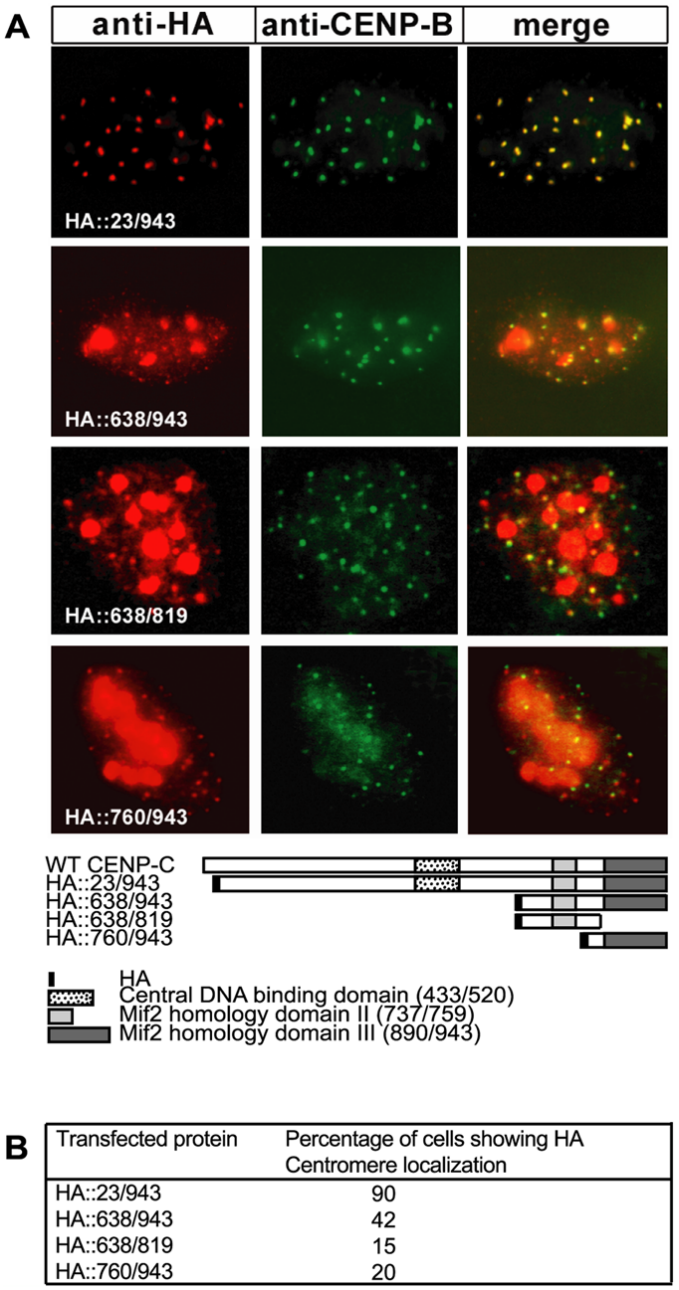
Both Mif2p homology domains II and III target centromeres. (A) The indicated chimeric HA::CENP-C proteins were expressed in human HEK-293T cells and revealed by an anti-HA monoclonal antibody (red signal), while endogenous CENP-B was detected with an anti-CENP-B polyclonal antibody (green signal). Co-localization of the HA::CENP-C proteins and CENP-B is shown in yellow in the merged image. In the diagram, bars describe the different CENP-C truncated proteins as compared to the wt protein; Mif2p homology domain II (light grey), Mif2p homology domain III (dark grey), central DNA binding domain (dotted box), HA-tag (black box). Localization of proteins within the nuclei has been determined by epifluorescent microscopy. (B) Centromere localization of HA::CENP-C fusion proteins in interphase nuclei. For each mutant at lest 100 transfected cells were scored for colocalization of HA and CENP-B signals.

We have previously shown that CENP-C contains two domains, the central and the C-terminal domain, that can independently target the human centromere and associate with the centromeric DNA. In vitro studies have shown that the C-terminal domain also contains a dimerization region that can induce formation of higher order structure of CENP-C [25]. To establish which portion of the C-terminal domain carries the DNA binding and dimerization activities, three constructs expressing HA-tagged derivatives of the human CENP-C regions, 638/943 (Mif2p II+III domains), 638/819 (Mif2p homology domain II) and 760–943 (Mif2p homology domain III) respectively (Figure 2), were generated and used in four different assays.

First, we tested the ability of the different truncated proteins to localize at centromeres. The mutated proteins were expressed in HEK-293 cells and analyzed for centromere localization by an immunofluorescence assay. As centromeric markers, we used endogenous CENP-B signals, since it is well-known that this sequence-specific DNA-binding protein localizes to the centromeric heterochromatin beneath the kinetochore [27], [28]. As positive controls, we used the HA::23/943 and HA::638/943 polypeptides that we previously showed to efficiently colocalize with the centromeric protein CENP-B in transfected nuclei [13]. HEK-293 cells were seeded on coverslips, separately transfected with the specific constructs and after 24 hours fixed and immunolabelled with anti-HA antibodies to detect the localization of the expressed proteins. Centromeres were marked with anti-CENP-B polyclonal antibodies. Figure 2A shows an example of the typical co-localization of HA proteins with the CENP-B marker. Efficiency of centromere targeting was determined as percentage of nuclei in which mutant proteins and CENP-B were observed to colocalize (Figure 2B). Results show that HA::638/819 and HA::760–943 were almost equally efficient in targeting centromeres, although they were both less efficient than a construct corresponding to the entire C-terminal domain.

**Figure 3:**
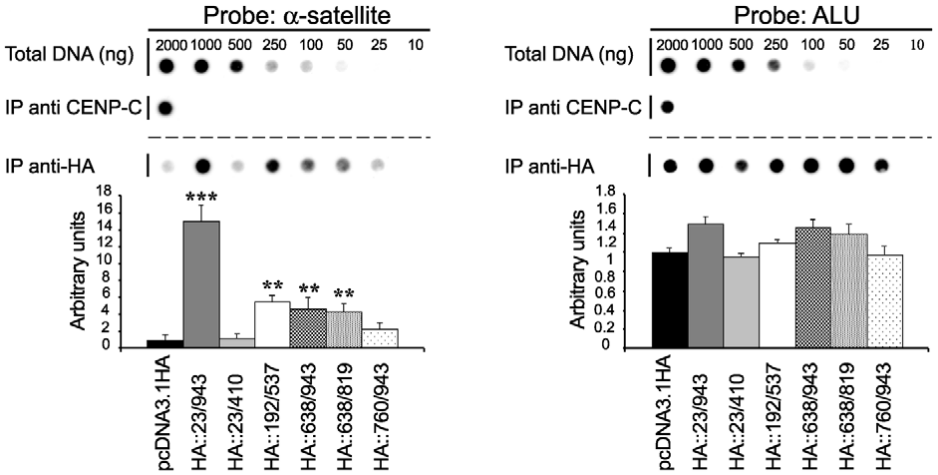
The Mif2p homology domain II of CENP-C binds the alpha satellite DNA in vivo. HA::CENP-C constructs were independently transfected into HEK-293T cells and their binding to alpha satellite DNA was analyzed by ChIP assay. Chromatin was immunoprecipitated by using anti-HA (IP anti-HA) or anti-CENP-C antibodies (IP anti-CENP-C). Immunoprecipitated DNAs along with total DNA were hybridized with alpha-satellite or Alu DNA probes. The relative enrichment of alpha-satellite DNA (left panel) was normalized to that of Alu sequences (right panel) and compared to that obtained from cells transfected with the empty vector, pcDNA3.1HA (IP background). Values are the mean±s.e.m. of at least three experiments.***P<0,01; **P<0,1.

Second, to understand which part of the C-terminal region is involved in centromere DNA recognition, the three HA-constructs were used in a Chromatin immunoprecipitation assay. We performed a formaldehyde crosslinking technique (ChIP) that was previously employed to demonstrate direct interaction of endogenous CENP-C to the alpha-satellite DNA [12]. Immunoprecipitated DNA was purified and transferred onto a nylon filter along with a scale of the input DNA. The filter was hybridized with a ^32^P-labelled alpha satellite specific probe. Hybridization signals above that of the control (pcDNA3.1HA) were interpreted as a significant enrichment of alpha satellite DNA. Signals generated by the alpha satellite probe were quantified by densitometric analysis and normalized to those obtained with an Alu sequence as previously described [13]. As shown in Figure 3, results indicate that HA::638/943 and HA::638/819 proteins can associate with alpha satellite DNA, although less efficiently than the full length CENP-C protein. On the contrary, the HA::760/943 protein cannot associate with the alpha satellite DNA with the same strength. These results suggest that the region between aa 638 and 759, containing the Mif2p homology domain II, is required for the ability of the C-terminal domain to bind the alpha satellite DNA.

### The Mif2p homology domain III can induce CENP-C dimerization/oligomerization

**Figure 4:**
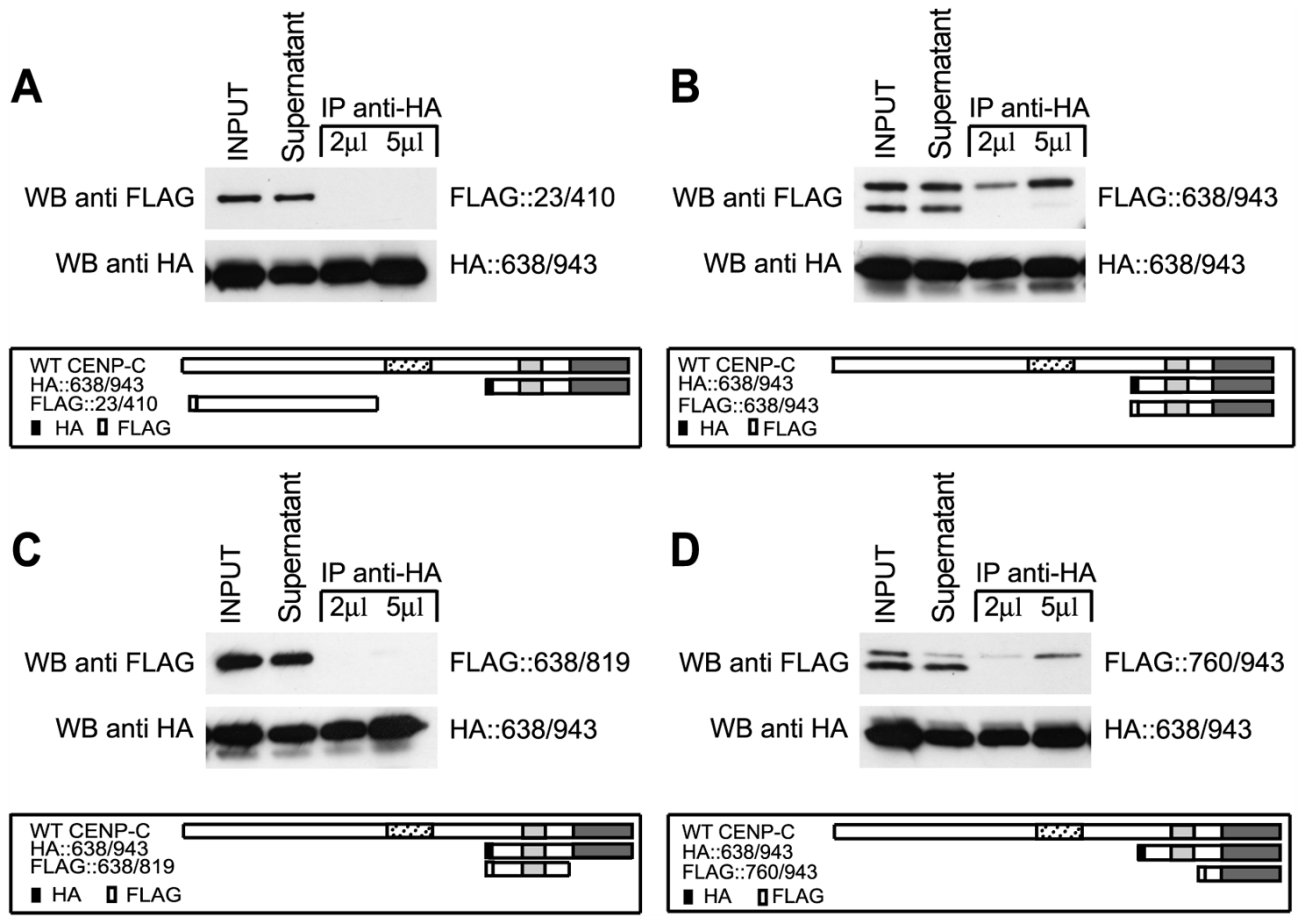
The Mif2p homology domain III of CENP-C possesses a self-associating activity in vivo. Cells HEK-293T were transiently transfected with the entire C-terminal region of CENP-C (HA::638/943) and FLAG::CENP-C constructs as indicated in the diagrams (A, FLAG::23/410; B, FLAG::638/943; C, FLAG::638/819 and D, FLAG::760/943). After 24 hours of expression, nuclear extracts were performed and proteins were immunoprecipitated through an anti-HA affinity matrix. Interactions between CENP-C domains are revealed by the presence of FLAG-tagged proteins in the IP anti-HA sample. The diagram in each panel illustrates the HA and FLAG CENP-C derivatives as compared to the full length protein: Mif2p homology domain II (light grey), Mif2p homology domain III (dark grey), central DNA binding domain (dotted box), HA-tag (black box), FLAG-tag (white box).

**Figure 5:**
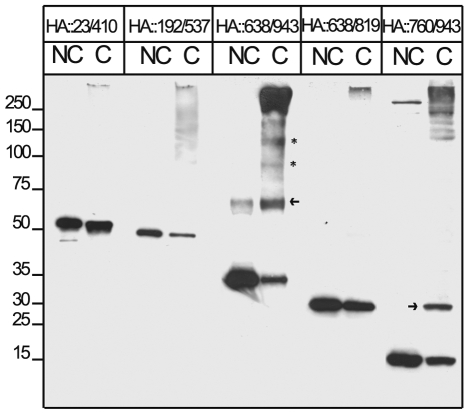
C-terminal domain of CENP-C can form dimers/oligomers in vitro. HA CENP-C truncated proteins were expressed in HEK-293T cells and immunopurified with anti-HA affinity matrix. Purified proteins were recovered by elution with an HA peptide and crosslinked (C) or not (NC) with glutaraldehyde to stabilize possible protein aggregates. Formation of possible oligomers was determined by western blot probed with an anti-HA antibody. Dimers are indicated by arrows whereas trimers and tetramers are indicated by asterisks.

Then we investigated the function of the Mif2p homology domain III located within the 760/943 region. One possibility is that this domain may be required to sustain CENP-C dimerization as inferred by previous studies [25]. To address this point, truncated CENP-C proteins labelled with HA- or FLAG-tag, as described in the scheme in Figure 4, were co-transfected in HEK-293 cells. Cells were lysed, nuclear extracts were prepared and subjected to immunoprecipitation with anti-HA antibodies. IP-protein complexes were analyzed by Western blotting using anti-FLAG antibodies. Detection of FLAG-tagged proteins in the IP-anti-HA sample lane indicates that the HA- and FLAG-labelled proteins can interact possibly by forming dimers or oligomers. Figure 4 shows that the FLAG::638/943 and FLAG::760/943 CENP-C polypeptides were efficiently co-immunoprecipitated with the C-terminal region of CENP-C (Figures 4B,D). In contrast the FLAG::638/819 polypeptide along with the negative control (FLAG::23/410) could not be co-immunoprecipitated (Figures 4A,C). This finding suggests that the last 120 aa of the CENP-C terminal end are critical for CENP-C self-association. To further investigate the nature of the association, HA-proteins were overexpressed in HEK-293 cells, purified through an anti-HA affinity column and eluted with an HA peptide used as a competitor. Eluted proteins were tested for oligomerization in vitro by using glutaraldehyde as a cross-linking agent in order to stabilize possible CENP-C oligomers. Next, cross-linked proteins were denatured in Laemmli sample buffer and separated by SDS-PAGE. CENP-C dimers/oligomers were detected by western blot using anti-HA polyclonal antibodies. The N-terminal end moiety of CENP-C (construct 23/410) and the central domain (construct 192/537) were also tested and used as negative controls. Results show that the HA::638/943 and HA::760/943 proteins can specifically form dimers, (as indicated by arrows in Figure 5) and higher molecular weight oligomers (see asterisks in Figure 5). In contrast the HA::638/819 protein as well as N-terminal (HA::23/410) and central (HA::192/534) CENP-C domains cannot form dimers/oligomers. Our data indicate that the region 820–943 containing the Mif2p homology domain III is required for CENP-C dimerization/oligomerization.

Taken together our findings reveal that the C-terminal end of CENP-C displays multiple functions including the ability to associate with the alpha satellite DNA and form higher order complexes.

### CENP-C interacts with CENP-A and histone H3

Several lines of evidence have suggested that CENP-C may interact with CENP-A. Specifically this point has been well highlighted by several papers in recent years [5], [29]–[31]. Based on these previous studies we decided to determine whether CENP-A directly interacts with CENP-C and particularly which protein domains are required for such interaction.

**Figure 6:**
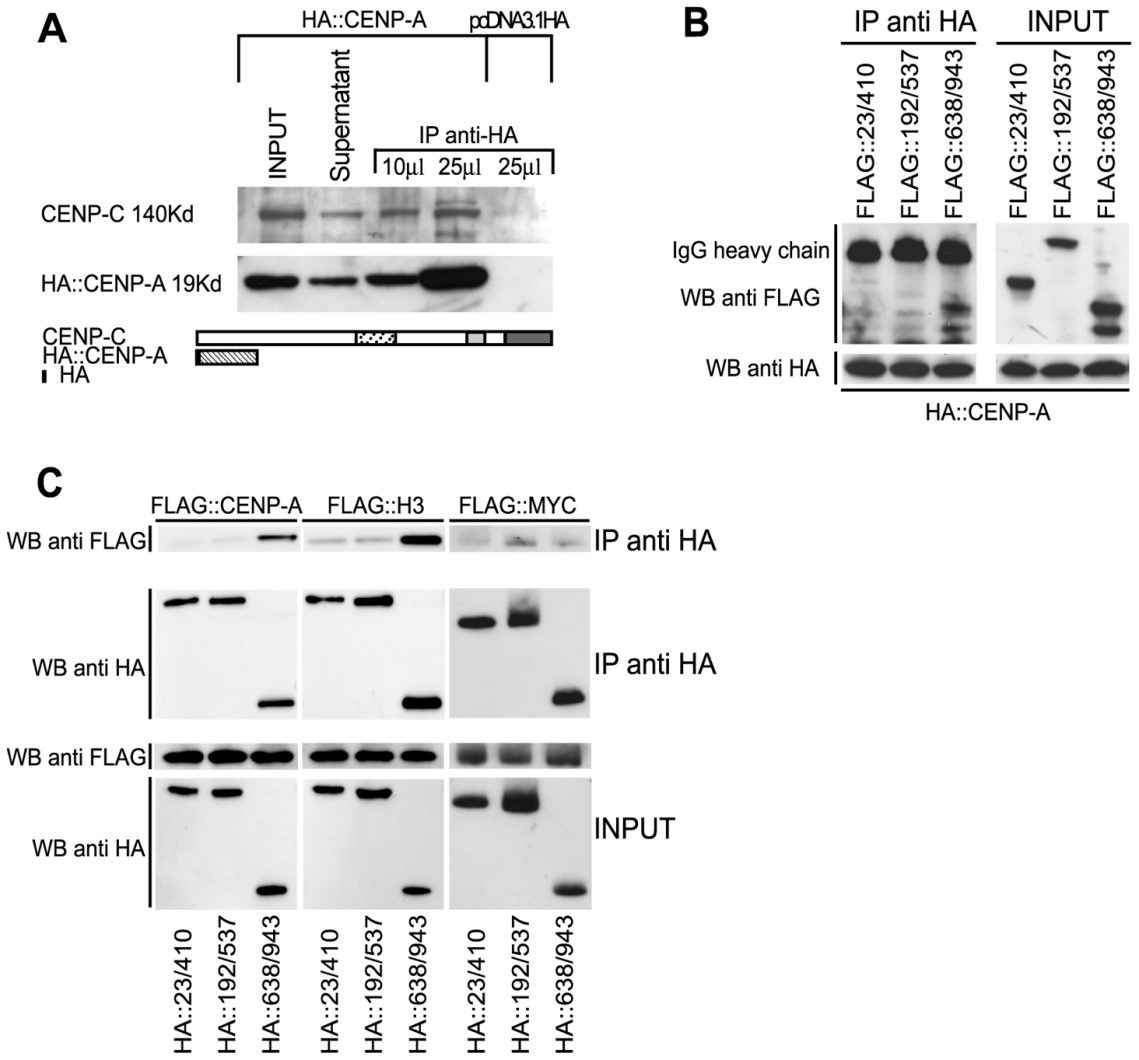
CENP-C can interact with CENP-A and histone H3 through its C-terminal domain. (A) HA::CENP-A coimmunoprecipitates the endogenous CENP-C. HEK-293 cells were transfected with HA::CENP-A or with the HA empty vector (pcDNA3.1HA) and HA proteins were purified from nuclear extracts with anti-HA affinity matrix. The presence of the endogenous CENP-C in the immunoprecipitated samples was determined by western blot with an anti-CENP-C antibody (B) Overexpressed HA::CENP-A coimmunoprecipitates FLAG C-terminal CENP-C domain. (C) Coimmunoprecipitation of overexpressed HA::CENP-C domains along with FLAG-tagged histone proteins and c-Myc protein. Only the construct that contains the C-terminal CENP-C region is able to interact with both CENP-A and histone H3.

To address this issue we looked for the formation of CENP-C/CENP-A complexes by a co-immunoprecipitation assay. A construct expressing an HA::CENP-A protein was transfected into HEK-293 cells and nuclear extracts were prepared and subjected to immunoprecipitation with anti-HA antibodies. IP-protein complexes were analyzed by Western blotting using polyclonal anti-CENP-C antibodies. As shown in Figure 6A, CENP-A can co-immunoprecipitate with endogenous CENP-C protein, suggesting that CENP-A and CENP-C can interact. Next, we determined which portion of CENP-C is required for this interaction. To do this, HEK-293 cells were transfected with the HA::CENP-A expression plasmid along with FLAG::CENP-C constructs expressing different portions of the protein (Figure 6B). Again the HA::CENP-A was immunoprecipitated and the IP complexes were analyzed by Western blotting with an anti-FLAG antibody. Results show that the C-terminal domain (aa 638–943) can be efficiently co-immunoprecipitated with CENP-A. Consistent results were obtained when the reciprocal experiment was performed. HA::CENP-C and FLAG::CENP-A constructs were cotransfected into HEK-293 cells, immunoprecipitated with an anti-HA antibody and FLAG-tagged proteins were detected (Figure 6C). Collectively these findings support the view that the C-terminal domain of CENP-C is required to contact CENP-A possibly at centromeres.

**Figure 7:**
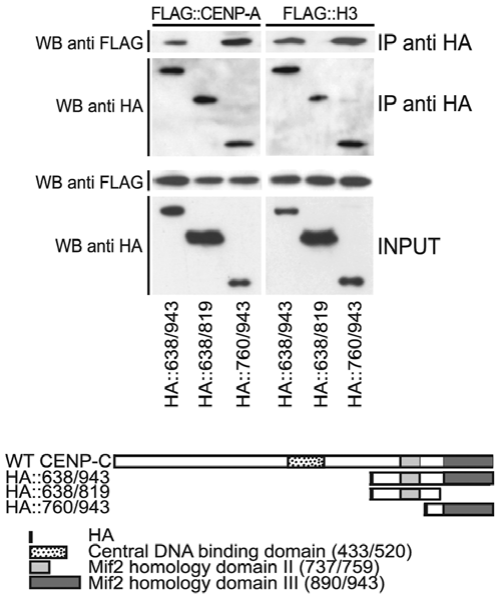
The Mif2p homology domain III mediates the interaction of CENP-C with CENP-A and histone H3. Coimmunoprecipitation of overexpressed HA::CENP-C C-terminal domains along with FLAG-tagged CENP-A and histone H3. Constructs containing the Mif2 homology domain III shows the ability to interact with both CENP-A and histone H3.

Considering the high level of homology between CENP-A and histone H3, we extended our analysis to evaluate the ability of CENP-C to interact with the histone protein H3 and with the transcription factor c-Myc, used as a negative control. FLAG-tagged proteins were co-expressed with the HA-CENP-C domains and the interaction was determined by co-immunoprecipitation assay. The results of the experiments, shown in Figure 6C, indicate that the C-terminal CENP-C domain interacts with histone H3, in addition to CENP-A, while the transcription factor c-Myc was not immunoprecipitated by CENP-C protein fragments. To further investigate which portion of the C-terminal region of CENP-C can mediate the interaction observed with CENP-A and histone H3, we performed a co-immunoprecipitation assay using FLAG tagged histones co-expressed along with the HA-CENP-C constructs obtained from the partitioning of C-terminal region (Figure 7). IP complexes were analyzed by Western blotting with an anti-FLAG antibody. Our results show that the Mif2p homology domain III contained in the HA::760/943 CENP-C construct is the only one able to interact both with CENP-A and H3.

### Interaction between CENP-C and CENP-A takes place at centromeres

**Figure 8:**
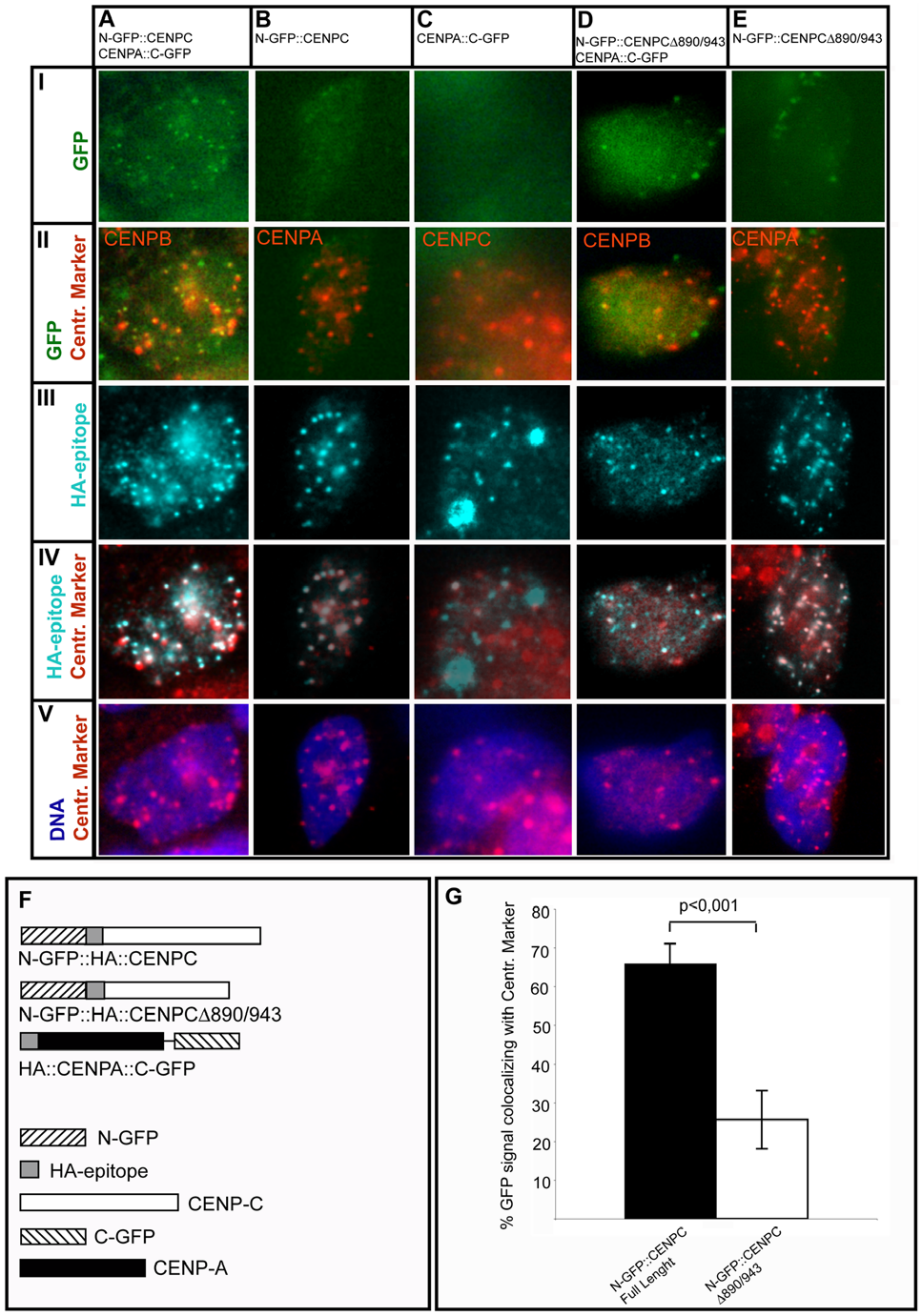
Interaction between CENP-C and CENP-A takes place at centromere positions. HEK293 cells transfected with plasmids expressing the chimeric protein combinations indicated at the top of each column A–E (A: N-GFP::HA::CENPC and HA::CENPA::C-GFP; B: N-GFP::HA::CENPC; C: HA::CENPA::C-GFP; D: N-GFP::HA::CENPCΔ890-943 and HA::CENPA::C-GFP; E: N-GFP::HA::CENPCΔ890-943). After fixation cells were immunolabelled with a monoclonal anti-HA antibody and polyclonal antibodies used as centromere markers (A and D: anti-CENP-B; B and E anti-CENP-A; C: anti-CENP-C). Row I shows complementing GFP (green); row II shows merge between complementing GFP and the centromeric marker (red); row III shows HA-tagged chimeric proteins (cyan); row IV shows merge between HA-tagged chimeric proteins and the centromeric marker; row V shows merge between centromeric marker and DNA (blue). F: diagrams of the chimeric proteins expressed in cells. G: quantification of colocalization between the green signal due to complementing GFP and the centromeric marker in cells that coexpress HA::CENPA::C-GFP and N-GFP::HA::CENPC full length or the truncated mutant N-GFP::HA::CENPCΔ890-943. 10 to 15 nuclei for each experiment were analyzed and the data expressed as ratio between GFP signal corresponding to centromeres and number of visible centromeres. Statistical significance have been checked with T-student test and indicated in the graph. The bright green punctate signals in panel AI indicate efficient GFP complementation in cells coexpressing N-GFP::HA::CENPC and HA::CENPA::C-GFP. The green signals in AI well correspond to centromere positions as indicated by merge in panel AII and to HA-tagged proteins shown in panels AIII-IV. The green signal shown by cells expressing respectively only N-GFP::HA::CENPC or HA::CENPA::C-GFP is very low (BI) or absent (CI) although the chimeric HA-tagged proteins are expressed. In cells expressing N-GFP::A::CENPCΔ890-943 and HA::CENPA::C-GFP few green signals are visible corresponding to centromere position (green and red in DI–II) suggesting lower interaction when CENP-C Mif2 homology domain III is deleted although N-GFP::HA::CENPCΔ890-943 localizes at centromeres (EIII–IV).

Since we could not exclude that some results of the CoIP experiments may be influenced by the presence of long centromeric chromatin in the nuclear extract, we decided to assess in vivo if the interaction between CENP-A and CENP-C takes place at human centromeres. To this purpose we adopted the Bimolecular Fluorescence Complementation (BiFC) assay. The technique is based on the observation that a fluorescent protein (in this work Enhanced-GFP) can be splitted in two fragments no longer able to emit a bright fluorescence. If the two fragments, fused to proteins that physically interact, are brought in close proximity they can functionally complement and emit bright fluorescence [32]–[36]. Moreover BiFC let to highlight where the protein-protein interaction takes place within the cell. Notably this approach has been successfully used to show protein-protein interaction involving histone-like proteins [37], [38]. To this purpose plasmids have been created to express: i) the HA::CENP-C protein fused at the HA epitope with the GFP N-terminal half (N-GFP::HA::CENPC) or with the C-terminal half (C-GFP::HA::CENPC), and ii) the HA::CENP-A protein fused at the C-terminal with the GFP C-terminal (HA::CENPA::C-GFP) or N-terminal halves (HA::CENPA::N-GFP). The four expression plasmids have let us to perform the BiFC assay by coexpressing two different chimeric protein combinations: 1) N-GFP::HA::CENPC/HA::CENPA::C-GFP and 2) C-GFP::HA::CENPC/HA::CENPA::N-GFP. Since the two sets of experiments produced very similar results we will describe in detail only the data obtained coexpressing N-GFP::HA::CENPC and HA::CENPA::C-GFP chimeric derivatives. HEK-293 cells where transiently transfected to express the two chimeric proteins either together or individually as negative controls. After 24 hours cells have been immunolabelled with a mouse-monoclonal anti-HA antibody to monitor the chimeric proteins subcellular localization and with polyclonal antibodies raised in rabbit to visualize, in interphase cells, an endogenously expressed centromeric protein used as centromere marker (anti-CENP-B in double transfection experiment; anti-CENP-A in N-GFP::HA::CENPC single transfection; anti-CENP-C in HA::CENPA::C-GFP single transfection). To monitor the green GFP emission due to fluorescence complementation, the described primary antibodies were revealed with secondary antibodies conjugated with the Cy5 and Cy3 fluorochromes whose fluorescence emission is respectively in the far-red and red wavelength range. As shown in figure 8 we could observe a punctate pattern due to bright green fluorescence only in cells that coexpress the CENP-C and the CENP-A derivatives (figure 8AI) but not in cells that express either one or the other chimeric protein (figure 8BI). This result indicates that fluorescence complementation can arise only when both N-GFP::HA::CENPC and HA::CENPA::C-GFP proteins are present in the same cell confirming our previous results that support a CENP-C/CENP-A direct interaction (Figures 6,7). Moreover we assessed that the two proteins interact at centromeres observing that the GFP punctate pattern (Figures 8AI) and the centromere marker position (Figures 8AII, 8AV) colocalize as revealed by merged patterns (Figures 8AII): in detail 65,9% of the green dots colocalize with the centromere marker (Figure 8G). To check if the CENP-C Mif2p homology domain III is indeed required for the CENP-C/CENP-A interaction at centromeres, we have built a plasmid to express a N-GFP::HA::CENPC derivative that lacks aa 890/943 (N-GFP::HA::CENPCΔ890/943). Following the above described procedure we have performed a BiFC assay coexpressing N-GFP::HA::CENPCΔ890/943 and HA::CENPA::C-GFP. As shown in Figure 8DI we could observe only few green dots localizing at centromere positions (Figure 8DI–II) indicating a lowered efficiency level of the CENP-C/CENP-A interaction. In detail in this experiment only 25,6% of the green signal correspond to centromere positions (Figure 8G).

## Discussion

In a previous study we have demonstrated that CENP-C contains two regions, the central one and the C-terminal one, that can both independently localize at the centromere and associate with alpha-satellite DNA [13]. Here, we have extended our previous results by further dissecting the C-terminal region of CENP-C and defining specific functions for the highly conserved Mif2p homology domains. Computational analysis of CENP-C protein sequences from different species reveals that the C-terminal Mif2p homology domains of CENP-C are strongly conserved during evolution; indeed, this region displays more than 80% amino acid identity between human and mouse [23], [24], [39]. This observation suggests that these domains must have been under negative selective pressure possibly to preserve an essential role for centromere function from yeast to mammals. Until now the specific role of the Mif2p homology domains of the C-terminal region have remained elusive. Our results show that the two regions display multiple and complex functions. First, ChIP experiments show that the Mif2p homology domain II is able to associate with the alpha satellite DNA and assign a specific function to this region. This result provides evidence for previous speculations, which proposed that this region might correspond to a putative DNA binding domain based on its specific amino acid content [23] and by the observations that the C-terminal region of CENP-C (aa 638–943) could weakly bind DNA in vitro [40]. These data are also consistent with previous observations showing that mutations in the Mif2p homology domain II of yeast, *Drosophila* and chicken can respectively, impair the proper structuring of the centromere [24], [41], abolish centromere localization [42], and induce metaphase delay and chromosome mis-segregation [43]. It should be mentioned that the CENP-C motif, at least for the yeast protein, has been described as containing acidic and proline-rich tracts typically found in the mammalian HMGI(Y) proteins [44], [45]. HMGI(Y) factors are chromatin structural components characterized by the ability to bind DNA without sequence specificity, probably recognizing particular DNA structures, as four-way (Holliday) junction [46]. This could be the case also for the Mif2p homology domain II considering that CENP-C, as well as CENP-A and PARP, was found to associate with active neocentromeres that apparently do not contain repetitive sequences, typical of conventional centromeres, suggesting that their localization to centromere may occur through epigenetic mechanisms [20], [47]–[49].

Second, our results indicate that the Mif2p homology domain III, corresponding to residues 890–943, is required for CENP-C dimerization and/or oligomerization. This finding is consistent with what has been proposed by Sugimoto and colleagues [25], and extends this previous study by showing that the Mif2p homology domain III is required to sustain dimer/oligomer formation in vivo. Unlike Sugimoto, we were unable to find evidences of oligomers formation with N-terminal or central CENP-C domains. As previously proposed for the centromere heterochromatin antigen CENP-B [50], the DNA binding and self-associating activities of CENP-C could contribute to the packaging of kinetochore DNA into centromere chromatin. Notably, a very recently published paper supports our data showing that in *S.cerevisiae* Mif2p protein the two domains corresponding to human CENP-C Mif2p homology domains II and III display DNA-binding activity and dimerization ability, respectively, and both of them are required for an efficient yeast growth [51].

Third, we show that the C-terminal region of CENP-C can physically interacts with CENP-A. This is an important result since it provides a mechanistic explanation for previous correlative studies showing that the centromere localization of CENP-C is strictly dependent on the presence of CENP-A [7], [8], [29], [52]. Since the dependence of CENP-C on CENP-A for targeting to centromere appears to be a general rule, one would expect that conserved domains could mediate the interaction between these proteins. Indeed, using both coimmunoprecipitation and bimolecular fluorescence complementation assays, we show that the C-terminal region of CENP-C, containing the evolutionarily conserved Mif2p homology domains III, is required for the interaction with CENP-A at the centromere position. It is possible that other proteins may participate CENP-C loading to centromere; for example it has been shown that Mif2p homology domains can interact with CENP-B [53]. Nonetheless, it seems unlikely that this interaction may represent the principal mechanism by which CENP-C is recruited to centromere considering that CENP-B gene inactivation does not affect kinetochore assembly [54] and that CENP-B is absent from the Y chromosome centromere or in neocentromeres where CENP-C is instead correctly localized [20], [47]–[49].

Another important finding reported here is the interaction between the Mif2p homology domain III on the C-terminal region of CENP-C and the histone H3 protein. This result, as well as the observation of a requirement of the same CENP-C domain for the association with CENP-A are supported by two papers published while this article was under revision [30], [31]. In these two papers the authors, using different approaches and different cell systems, strongly argue in favour of CENP-C being able to associate both with CENP-A and histone H3. In addition, another recent study suggests that a subset of heterotypic nucleosomes containing both histone H3 and CENP-A may exist in centromeric chromatin [5]. Moreover, Blower and colleagues have identified blocks of CENP-A and histone H3 nucleosomes linearly interspersed in human and *Drosophila* centromeric chromatin [55]. A similar organization is present in neocentromere where H3-associated nucleosomes are present at the intervening region between the CENP-A-binding clusters [56]. On the basis of these observations it has been proposed that the CENP-A containing chromatin is organized along the exterior surface of each sister chromatid whereas interior surface is occupied by H3-containing nucleosomes ([55], [57], [58]). Our findings extend this view and would allow us to speculate that CENP-C, by virtue of its ability to establish multiple contacts with DNA and centromere proteins, may serve to structure and/or stabilize the nucleosomal blocks of the interior and exterior surfaces of each sister chromatids. Nonetheless structural studies are needed to investigate this hypothesis.

Overall this study assigns specific functions to the conserved Mif2p homology domains present into the human CENP-C and provides support to a structuring role of CENP-C in the organization of centromeric chromatin.

## Materials and Methods

### DNA Constructs

Full length CENP-C and CENP-A cDNAs were amplified by RT-PCR from HeLa cells and cloned into the pCDNA3.1 vector and in frame with the HA epitope tag. CENP-A primers: 1F: 5′-AAAGGGAGATCTGGCCCGCGCCGCCGGAGCCG-3′ and 140R: 5′-AAAGGGGAATTCTCAGCCGAGTCCCTCCTCAAGG-3′. HA-tagged full length CENP-C and carboxyl or/and N-terminal deletion mutants of CENP-C were generated by PCR as previously described [13] except for the HA::638/819 and HA::760/943. The HA::638/819 mutant has been generated by PCR using the following oligonucleotides as primers: 638F: 5′-AAAGGGGGATCCAGAAGCTCAAAGAATGAAG-3′ and 819R: 5′-AAAGGGGAATTCTCATCCAAGAGGTATACCTAG-3′. The HA::760/943 mutant has been generated by PCR using the following oligonucleotides as primers: 760F: 5′-AAAGGGGGATTCGGAAGGCCATCAGGAGGATTCG-3′ and 943R: 5′-AAAGGGGAATTCTCATCTTTTTATCTGAGTAAAAAG-3′. FLAG-tagged C- and/or N-terminal deletion mutants of CENP-C were generated by PCR; the oligonucleotide sequences used as primers to generate the different expression plasmids are listed below: i) 23/410: 23Fh: 5′-AAAGGGAAGCTTGCACGTGACATTAACACAGAG-3′ and 410R: 5′-AAAGGGGAATTCTCATGGTTTTCTGCATTCTTGG-3′; ii) 638/943: 638Fh: 5′-AAAGGGAAGCTTAGAAGCTCAAAGAATGAAG-3′ and 943R iii) 638/819: 638Fh and 819R iv) 760/943: 760Fh: 5′-AAAGGGAAGCTTGGAAGGCCATCAGGAGGATTCG-3′ and 943R. PCR products were digested with HindIII and EcoRI and directionally cloned into the 3xFLAGpCMV10 vector (Sigma Chemical Co., St. Louis, Mo.) in frame with the N-terminal FLAG-tag. Full length histone H3 cDNA was amplified by RT-PCR from HeLa cells and cloned into the 3xFLAGpCMV10 vector (Sigma). Histone H3 primers: 1F3: 5′-AAAGGGAAGCTTGCCCGAACCAAGCAGACTGCGCGCAAG-3′ and 136R 5′-AAAGGGGAATTCTCAGGCCCGCTCCCCGCGGATAC-3′. All the constructs were verified by sequencing.

The expression plasmids to perform the Bimolecular Fluorescence Complementation (BiFC) assay have been built as described below. To build N-GFP::HA::CENPC and C-GFP::HA::CENPC expression plasmids, the Enhanced-GFP N-terminal (N-GFP, aa 1/157) and the C-Terminal (C-GFP, aa 158/239) halves were generated by PCR using as template the pEGFP-C3 vector (Clontech, GenBank Acc.: U57607) and subcloned into the HA-tagged CENP-C full length construct previously described. The HA-epitope has been left as flexible linker between the GFP derived fragment and CENP-C. The oligonucleotide sequences used as primers and the cloning procedures to generate the different expression plasmids are described below: i) full length N-GFP::HA::CENPC: NGFPCENPCH3F: 5′-GGGAAGCTTATGGTGAGCAAGGG-3′ and NGFPCENPCH3R: 5′-GGGAAGCTTCTTGTCGGCCATG-3′. The PCR products were digested with HindIII and cloned into pCDNA3.1/HA-CENPC after HindIII digestion at a HindIII target site placed 5′ with the respect to the HA epitope ATG codon and in frame with the N-terminal HA-tag. The correct orientation of the cloned N-GFP fragment has been evaluated by PCR assay using a CENP-C internal reverse primer. ii) full length C-GFP::HA::CENPC: CGFPCENPCF: 5′-ATGCAGAAGAACGGCATCAAG-3′ and CGFPCENPCR: 5′-TGTTATCTAGATCCGGTGGATCC-3′. PCR products were phosphorylated at the 5′-OH terminals with T4 polynucleotide Kinase (New England Biolabs) following manufacturer instructions and blunt-end ligated in pCDN3.1/HA::CENPC::23/943 after digestion at the Hind III site placed 5′ to the HA coding sequence and after fill-in standard procedure to make blunt HindIII protruding terminals. The correct orientation of the C-GFP fragment was evaluated on the base of BamHI digestion in the C-GFP fragment and downstream of the HA epitope coding sequence. The N-GFP::HA::CENPCΔ890/943 deletion mutant was generated by PCR starting from the described N-GFP::HA::CENPC full length construct using as primers the following oligonucleotides: NGFPCENPCPst1F: GGGCTGCAGACCATGGTGAGCAAGGG and CENPC889ER1R: CCGGAATTCTCATATATCCTGGCCAAC. The PCR products were digested with PstI and EcoRI and directionally cloned into the pCDNA3.1 vector.

The HA::CENPA::N-GFP and HA::CENPA::C-GFP expression plasmids were generated as described below and notably creating an aqueous soluble and flexible 2XGly-Ser-Ser-Ser linker between the CENP-A C-terminal end and the GFP fragments N-terminal end by introducing the linker coding sequence in the oligonucleotides used as primers for the PCR reactions. HA-CENPA coding sequence has been amplified by PCR starting from the described HA-CENPA plasmid and using as primers the following oligonucleotides: CENPASAL1F: 5′-AAAGTCGACATGGGCCCGCGCCGC-3′ and CENPALNKSPH1R: 5′-ATGCATGCGCTGCTGCTGCCGCCGAGTCCCTCCTC-3′. The PCR product was digested with SphI and SalI restriction enzymes. The N-GFP and C-GFP fragments were amplified by PCR using as template the pEGFP-C3 vector (Clontech, GenBank Acc.: U57607) and as primers the following oligonucleotides: i) for N-GFP amplification NGFPLNKSPH1F: 5′-ATGCATGCGGCAGCAGCAGCATGGTGAGCAAGGGC-3′ and NGFPER1R: TGAATTCTCACTTGTCGGCCATGATATAGACGTT; the PCR product was digested with SphI and EcoRI restriction enzymes; ii) for C-GFP amplification CGFPLNKSPH1F: 5′-ATGCATGCGGCAGCAGCAGCCAGAAGAACGGCATC-3′ and 5′-CGFPMFE1R: CTCCTCCAATTGCTCCTCTCAGTTATCTAGATCCGG-3′. The PCR product was digested with SphI and MfeI restriction enzymes. The MfeI restriction enzyme leaves protruding ends compatible with EcoRI terminal ends. The pairs of PCR products identified by the forward and reverse primer combinations listed below where added in the same ligation reaction and directionally cloned into pCDNA3.1 after digestion with EcoR1 and SalI restriction enzymes: i) CENPASAL1F/CENPALNKSPH1R and NGFPLNKSPH1F/NGFPER1R to build the HA::CENPA::N-GFP expression plasmid; the correct cloning was verified by HindIII and EcorI digestion at internal and MCS restriction sites; ii) CENPASAL1F/CENPALNKSPH1R and CGFPLNKSPH1F/CGFPMFE1R to build the HA::CENPA::C-GFP expression plasmid; the correct cloning was verified by HindIII digestion at internal restriction sites. All the above described plasmid constructs were verified by DNA sequencing and the molecular weight of the fusion proteins produced after transfection in cells has been checked by standard SDS-PAGE/Western-Blot procedure as described below using both anti-HA and anti-CENP-C or anti-CENP-A antibodies.

### Antibodies Production

Anti CENP-A antibodies: the DNA sequence encoding amino acids 1–40 of human CENP-A was cloned into pGEX2T vector and expressed in *E. coli* BL21 cells. GST-tagged recombinant protein was affinity purified by glutathione agarose beads and used to immunize two rabbits (387 and 386).

Anti CENP-C antibodies: the DNA sequence encoding amino-terminal half of human CENP-C (amino acids 23–410) was cloned into pFastBac HTb vector (Invitrogen). The histidine-tagged recombinant protein was expressed in SF9 cells using Bac-to-Bac baculovirus expression system (Invitrogen) and affinity purified by nickel column chromatography. The purified recombinant protein was used to immunize two rabbits (410 and 411).

Anti-CENP-A and anti-CENP-C sera were produced by the Eurogentec polyclonal antibody service. The antibodies specificity has been tested by Western-Blot, ChIP and Immuno Fluorescence.

### Immunocytolabelling

HEK293 cells (5×10^5^) were seeded on a collagen-coated coverslip and transfected with 3 µg of the different HA::CENP-C construct plasmid DNAs following the previously described method [13]. After 24 hours cells were rinsed in cold PBS 1X and incubated in 1X PBS/0,5% Triton X-100 for 2 min on ice to remove most of the cytoplasm (modified from [59]) then fixed for 10–15 min in 4% para-formaldehyde in 1X PBS, pH 7.0 at room temperature. Double immunolabelling were performed at room temperature for 90 min or at 4°C over night with pairs of the antibodies listed below: polyclonal rabbit anti-CENP-B antiserum 764 [60], polyclonal rabbit anti-CENP-C and anti-CENP-A antisera, monoclonal mouse anti-HA antibody (Santa Cruz Biotechnology). All the used antibodies where diluted at 1:1000 to 1:2000 in PBST (PBS1X, 0,1% Triton X-100) containing 4% normal goat serum as blocking reagent. After three 5 min washes with PBST, cells were incubated at room temperature with pairs of the secondary antibodies listed below: goat anti-mouse Cy3-conjugated, goat anti-rabbit FITC-conjugated, goat anti-rabbit Cy5-conjugated (Jackson Immuno Research) at a 1:400 dilution in 4% normal goat serum-PBST for 90 min. Finally nuclei were stained with Hoechst 33258 (Sigma) 2 µg/ml for 7 min, washed three times for 5 min in PBS1X and the coverlips mounted in Vectashield (Vector) or Prolong (Invitrogen) mounting media. The centromere localization of the HA::CENP-C fusion proteins and the BiFC assay analyses have been performed using a conventional epifluorescence microscope (Axioskop, Zeiss) equipped with a digital CCD camera (Axiocam, Zeiss) used for image capturing. For each HA::CENPC deletion mutant at least 100 transfected nuclei were scored for colocalization of HA and CENP-B signals. For the BiFC assay cells images were captured registering in grey-scale the emission signal in 4 different wavelength ranges: green from complementing GFP, far-red from anti-centromeric marker labelling, red from anti-HA labelling; ultraviolet from DNA. Image coloring and collages were made using Adobe Photoshop and Adobe Illustrator (Adobe System, Inc).

### Coimmunoprecipitation assay

HEK-293T cells (2×10^7^) were cotransfected with 10 µg of the two different HA/FLAG::CENP-C construct plasmids in equal ratio by means of the lipofectamine method (Roche). After 24 hours cells were harvested and resuspended in 5× cell volumes of hypotonic buffer (10 mM HEPES pH 8.0, 50 mM NaCl, 1 mM EDTA, 1 mM DTT, 1 mM PMSF, complete 1× protease inhibitor cocktail) allowed to swell on ice for 10 min. Cells were transferred to a glass Dounce homogenizer and homogenized through 25 up-and-down strokes using the tight pestle. Nuclei were collected by centrifugation, resuspended in 150 µl of high-salt buffer (20 mM HEPES pH 8.0, 420 mM NaCl, 1 mM EDTA, 1 mM DTT, 1 mM PMSF, complete 1× protease inhibitor cocktail, 10% glycerol) and incubated on a rocker for 30 min at 4°C. Nuclear extracts were recovered by centrifugation at 13.000 rpm for 15 min and quantified with BCA protein assay kit (Pierce). For each immunoprecipitation 1 mg of nuclear extract was diluted 1:1 with dilution buffer (1 mM EDTA, 1 mM DTT, 1 mM PMSF, complete 1× protease inhibitor cocktail, 10% glycerol) to lower the salt concentration and pre-cleared with Protein A agarose beads (Sigma) for 3 hours of incubation on a rocker; samples were recovered by centrifuging for 2 min at 2000 rpm in a chilled micro centrifuge. Precleared nuclear extracts were incubated with 50 µl of the anti-HA-affinity matrix (Sigma) and incubated overnight at 4°C on a rocker. Resin was washed 5 times with 1 ml of washing buffer (10 mM Tris-Cl, 140 mM NaCl, 0.5% Triton-X100, 0.5% Sodium deoxycholate, 1 mM DTT, 1 mM PMSF). Protein complexes were separated on a pre-cast 4–12% SDS-polyacrylamide gel (Invitrogen) and transferred onto a nitrocellulose membrane. The filter was blocked with TBS (150 mM NaCl, 25 mM Tris-Cl pH 8) and 4% nonfat dry milk and probed with a polyclonal anti-HA (Santa Cruz Biotechnology), or a polyclonal anti-CENP-C 410 or a monoclonal anti-FLAG (Sigma) antiserum: The filter was washed three times with TBST (TBS+0.1% Tween-20) and incubated with anti-rabbit or anti-mouse peroxidase-conjugated secondary antibodies (Amersham). Ig/protein complexes were revealed by ECL method (Amersham).

### Glutaraldehyde Cross-linking

HA::CENP-C mutants were purified with the anti-HA-affinity matrix (Sigma) as described above and eluted through 2 cycles of incubation with 50 µl of 1 mg/ml HA peptide. 2 µl of the purified protein were crosslinked in a solution containing 300 mM NaCl, 20 mM HEPES pH 8.0, 1% NP40, 2 µg BSA and 0.01% glutaraldehyde for 2 min at room temperature and blocked with 100 mM Tris-Cl pH 8. Crosslinked and uncrosslinked samples were analyzed by Western blotting.

### Formaldehyde Cross-linking and Chromatin/Immunoprecipitation Assay

ChIP was performed as described [61]. Constructs were separately transfected into HEK-293 cells and 24 hours later cells were treated with 1% formaldehyde and sonicated. Chromatin was then immunoprecipitated with anti HA antibodies and heat-treated for 6 hours at 65°C to reverse crosslink. Immunoprecipitations were performed with 5 µl of rabbit pre-immune serum (PI serum) or 5 µl of polyclonal antiserum against either CENP-C or HA (Santa Cruz Biotechnology). The immunoprecipitated DNA samples were analyzed by Dot Blot as described below.

### Dot blot analysis of immunoprecipitated DNA

Total input DNA and immunoprecipitated DNA samples were transferred to a Hybond-N^+^ filter (Amersham). The filter was pre-hybridized and hybridized with a solution containing 7% SDS and 0.5 M Na_3_PO_4_ pH 7.0. Probes were [α-^32^P]-labeled with the Megaprime kit (Amersham) and added to the hybridization solution at a specific activity of 2×10^9^ cpm/µg. Alpha-satellite DNA probes [62] used for the hybridization were: i) pZ7.6B (680 bp) detecting chromosome 7; ii) pZ21.A (850 bp) detecting chromosomes 13 and 21. The probe detecting Alu repeats [63] was: BLUR-8 (300 bp). Dot blot analysis was performed by transferring total input DNA and the immunoprecipitated DNA samples onto a Hybond-N^+^ filter (Amersham) with a Bio-Dot Apparatus (Biorad), as previously described [13]. ^***^ *P*<0,001; ^**^ *P*<0,01 (*t*-test).
